# Two Birds with One Stone: A Novel Dithiomaleimide-Based GalNAc-siRNA Conjugate Enabling Good siRNA Delivery and Traceability

**DOI:** 10.3390/molecules28207184

**Published:** 2023-10-19

**Authors:** Sudong Kong, Xiaoqing Gao, Qianhui Wang, Jianguo Lin, Ling Qiu, Minhao Xie

**Affiliations:** 1 School of Chemical and Material Engineering, Jiangnan University, Wuxi 214122, China; kongsd@bsyntech.com (S.K.); highlight25190@163.com (X.G.); 6220606073@stu.jiangnan.edu.cn (Q.W.); 2 NHC Key Laboratory of Nuclear Medicine, Jiangsu Key Laboratory of Molecular Nuclear Medicine, Jiangsu Institute of Nuclear Medicine, Wuxi 214063, China; linjianguo@jsinm.org; 3 Suzhou Biosyntech Co., Ltd., Suzhou 215300, China

**Keywords:** GalNAc-siRNA conjugate, siRNA delivery, dithiomaleimides, click chemistry

## Abstract

For the first time, a novel dithiomaleimides (DTM) based tetra-antennary GalNAc conjugate was developed, which enable both efficient siRNA delivery and good traceability, without incorporating extra fluorophores. This conjugate can be readily constructed by three click-type reactions, that is, amidations, thiol-dibromomaleimide addition and copper catalyzed azide–alkyne cycloaddition (CuAAC). And it also has comparable siRNA delivery efficiency, with a GalNAc L96 standard to *mTTR* target. Additionally, due to the internal DTMs, a highly fluorescent emission was observed, which benefited delivery tracking and reduced the cost and side effects of the extra addition of hydrophobic dye molecules. In all, the simple incorporation of DTMs to the GalNAc conjugate structure has potential in gene therapy and tracking applications.

## 1. Introduction

Synthetic small interfering RNAs (siRNAs) can inhibit the expression of ill genes through gene silencing when mediated by the endogenous RNA interference (RNAi) approach [1,2]. siRNAs have many advantages over small molecular and monoclonal antibodies because of their function of complete Watson–Crick base pairing with mRNA, whereas small molecule and monoclonal antibodies need to recognize the complicated conformation of proteins. Therefore, many diseases that are not treatable by small molecule and monoclonal antibodies since some target molecules cannot be identified. In contrast, any gene can be targeted by siRNA because only the right sequence along the targeting mRNA needs to be selected. As a result, siRNA has a wider therapeutic area than small molecules or antibodies [3,4,5,6,7]. However, efficient and targeted delivery remains the bottleneck, due to the large molecular weight and negative charge [8,9,10]. Recently, N-acetylgalactosamine (GalNAc) conjugates were successfully and widely applied for liver-targeted siRNA delivery due to their excellent interaction with the asialoglycoprotein receptor (ASGPR, also known as Ashwell-Morell receptor) [11,12,13]. By conjugating GalNAc to nucleic acid molecules, drug payloads can be efficiently delivered into hepatocytes and elicit the corresponding biological response. This represents a potent and promising delivery strategy for nucleic acid-based therapeutics. As a trailblazer in this research field, Alnylam Pharmaceuticals has made significant contributions on GalNAc conjugate based delivery system [14,15,16,17,18,19,20,21,22,23].

**Scheme 1:**
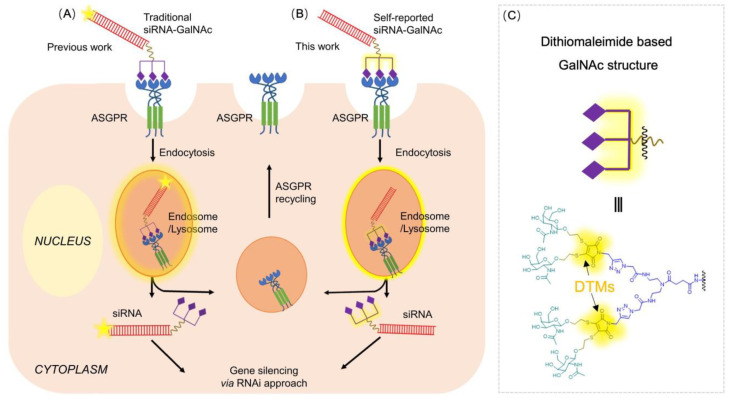
(**A**) General illustration of conventional GalNAc-decorated oligonucleotides (siRNA) delivery mechanism and tracking strategy, (**B**) The mechanism of our DTM based tetra-antennary GalNAc-siRNA conjugate without incorporating extra fluorophores, (**C**) The chemical structure of DTM based tetra-antennary GalNAc. For simplicity, only trivalent GalNAc conjugates are indicated.

A common issue for tracking the GalNAc delivery system should mandatorily incorporating fluorophore into the conjugate (Scheme 1A). As a result, it is often ambiguous regarding the location of the binding molecule and its mobility in the micellar host of the binding agent and also the payloads. Another problem arising from the conjugates aggregation could decrease the fluorescent emissions through either fluorophore-– interaction or fluorophore–fluorophore self-quenching events [24]. Furthermore, the incorporation of an emission handle, often a large hydrophobic dye molecule, such as fluorescein- and cyanine-type dyes, can lead to changes in the scaffold size and stability of the GalNAc conjugates [25]. Additionally, inducing extra fluorophore would increase the cost of the siRNA synthesis. Thus, new methods for the facile, economic and nondisruptive fluorophore labeling of GalNAc conjugates are required.

One of the reasonable solutions to these issues is the addition of a fluorophore, which can be readily and non-ambiguous incorporated into the GalNAc scaffold during the synthesis. Dithiomaleimides (DTMs) as a versatile and high-emission fluorophores, have been applied in protein and polymer labeling [26,27,28,29,30]. It could be prepared by a rapid and efficient reaction of 2,3-dibromomaleimide with two thiol motifs [31,32,33,34]. However, DTMs were also reported to exhibit a strong fluorescence property which can potentially act as a trackable and self-reported functional handle [35,36].

Herein, for the first time, we present a novel DTM-based tetra-antennary GalNAc conjugate, which enables both efficient siRNA delivery and good traceability without incorporating extra fluorophores (Scheme 1B,C). This conjugate can be readily constructed by three click-type reactions, that is, amidations, thiol-dibromomaleimide addition and copper catalyzed azide–alkyne cycloaddition (CuAAC). And, it also has comparable siRNA delivery efficiency with GalNAc L96 standard to *mTTR* target [15]. Additionally, due to the internal DTMs, a highly fluorescent emission was observed, which benefited the delivery tracking and reduced the cost and side effects of the extra addition of hydrophobic dye molecules. In all, the simple incorporation of DTMs inthe GalNAc conjugate structure could seem trivial, but we propose this new system has potential in gene therapy and tracking applications.

## 2. Results and Discussion

### 2.1. DTM GalNAc Ligand Synthesis

**Scheme 2:**
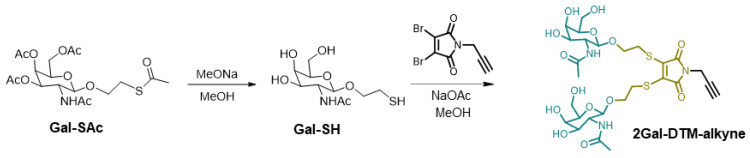
Synthesis of DTM-GalNAc ligand (2Gal-DTM-alkyne).

The DTM containing intermediate 2Gal-DTM-alkyne was synthesis as Scheme 2. Firstly, GalNAc containing the thiol group (Gal-SH) was first synthesized through the deprotection of acetyl protected Gal-SAc. Gal-SH was then conjugated to N-propargyl-2,3-dibromomaleimide in the presence of NaOAc as the catalyst to yield 2Gal-DTM-alkyne. The overall yield of these two-step reactions was around 80% (Scheme S1 and Figures S1–S5).

### 2.2. DTM-GalNAc-siRNA Conjugate Synthesis

**Figure 1:**
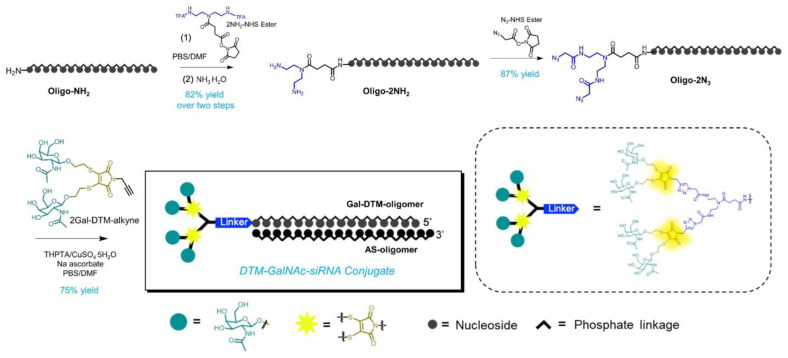
Synthetic route of the DTM-GalNAc-siRNA conjugate.

**Figure 2:**
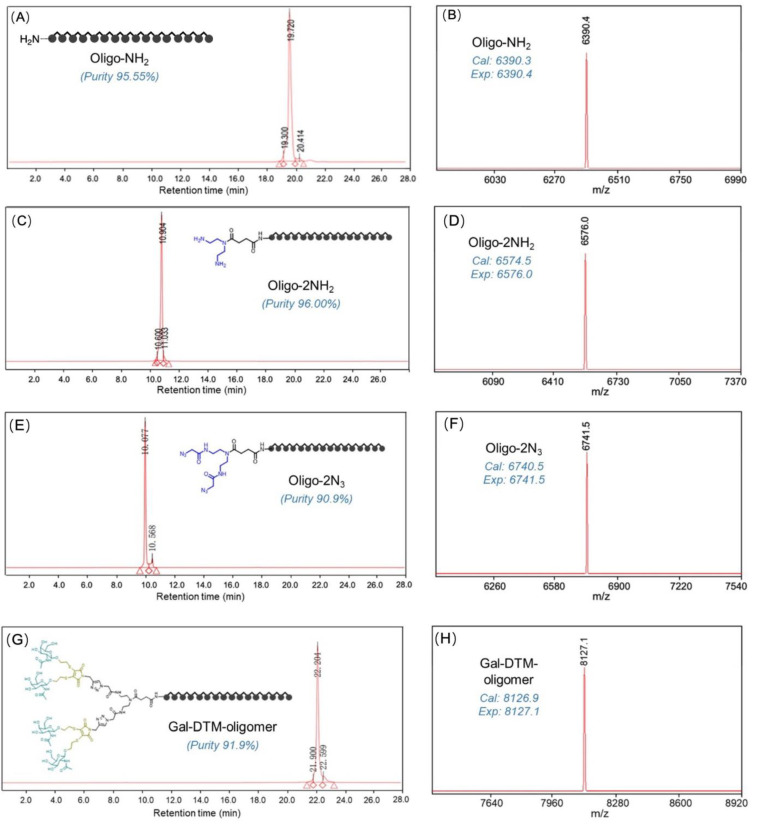
Structure and purity characterization: HPLC traces (**A**,**E**,**C**,**G**) and mass spectra (**B**,**D**,**F**,**H**).

As shown in Figure 1, the sense (oligo-NH_2_) and antisense (AS-oligomer) strands (Table 1) were synthesized, respectively on an ABI Synthesizer using commercially available 5′-*O*-(4,4′-dimethoxytrityl)-2′-deoxy-2′-fluoro-, 5′-*O*-(4,4′-dimethoxytrityl)-2′-*O*-(tert-butyldimethylsilyl)- and 5′-*O*-(4,4′-dimethoxytrityl)-2′-*O*-methyl-3′-*O*-(2-cyanoethyl-*N*,*N*-diisopropyl) phosphoramidite monomers of uridine and 4-*N*-acetylcytidine, 6-*N*-benzoyladenosine, and 2-*N*-isobutyrylguanosine using standard solid-phase oligonucleotide synthesis and deprotection protocols. Then, the sense strand (oligo-NH_2_) was successively coupled with two NHS linkers to equip two azido groups at the end (oligo-2N_3_) via efficient amidation reactions, in 82% and 87% of the yield, respectively. Oligo-2N_3_ was easily coupled with two 2Gal-DTM-alkyne via a CuAAc reaction to yield the tetra-antennary DTM-GalNAc-decorated sense strand in 75% of the yield (a detailed experimental methodology is listed in Section 3.2.2 in maintext). Moreover, the molecular structure and the purity of each oligomer was further validated by high performance liquid chromatography (HPLC) and electrospray ionization mass spectrometry (ESI-MS) (Figure 2A–H). Unimodal and symmetrical HPLC traces with high purity (90.9% to 96.0%), as well as single-peak signal, agree with the calculated value of the molecular mass, confirmed by the successful synthesis of the strands. Additionally, equimolar amounts of complementary sense and antisense strands were mixed and annealed by heating to 90 °C and slowly cooled to obtain the desired DTM-GalNAc-siRNA conjugate.

### 2.3. Cell Uptake of DTM-GalNAc-siRNA Conjugate

**Figure 3:**
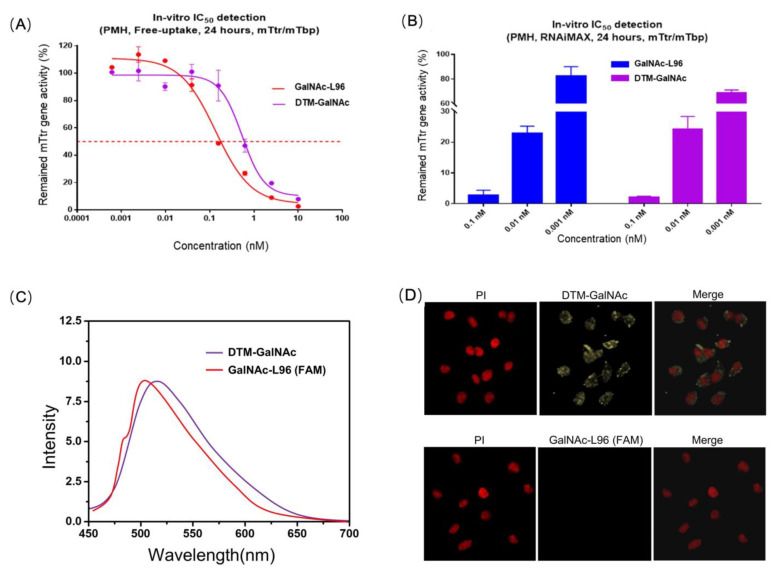
(**A**) Uptake of conjugates into primary mouse hepatocytes. Freshly isolated hepatocytes from livers of wild-type mice were incubated with 10 nM siRNA. (**B**) TTR gene silencing 24 h after transfection, samples were collected, and RNA was extracted with cell lysate added with RNase inhibitor. Kits were then used to remove genomic DNA and reverse transcription. The obtained cDNA was diluted and detected by fluorescence quantitative PCR. (**C**) Fluorescence spectra of DTM-GalNAc (purple line) and GalNAc-L96 (FAM) (red line). (**D**) CLSM images of hepatocytes incubated with FAM labeled antisense GalNAc-L96-siRNA conjugate (yellow), and label free DTM-GalNAc-siRNA conjugate (yellow) at an oligonucleotide concentration of 2 μm at 37 °C for 2 h. Cell nuclei (red) were stained with PI. (Scale bar: 20 μm).

The uptake of the siRNA-GalNAc conjugates was evaluated in freshly isolated primary mouse hepatocytes [37]. After cells were incubated with 10 nM siRNA, a comparable robust uptake was observed with standard GalNAc-L96 and our DTM-GalNAc (Figure 3A, part 3 in Supplementary Information). We next evaluated the ability to silence gene expression using GalNAc-L96 and our DTM-GalNAc, designed to target the rodent transthyretin (TTR) gene. The results of the fluorescence quantitative PCR also confirmed the comparable TTR gene silencing ability, especially in high concentrations (Figure 3B). As shown in Figure 3C, the DTM-GalNAc showed an excitation wavelength of 467 nm and an emission wavelength of 515 nm, which is similar to the fluorescence property of GalNAc-L96 (FAM). Moreover, for confocal laser scanning microscopy (CLSM), DTM-GalNAc-treated hepatocytes exhibited stronger green fluorescence in the cytoplasm than that of the FAM-labeled antisense GalNAc-L96-siRNA conjugate-treated group after 2 h incubation, as shown in Figure 3D. Overall, both IC_50_ detection and CLSM results indicated the efficient cellular uptake behavior of the obtained DTM-GalNAc, implying its potential to be an effective carrier for both good siRNA delivery and excellent traceability. The endosomal escape mechanism of DTM-GalNAc-siRNA conjugate is the same as the GalNAc-L96-siRNA conjugate. DTM-GalNAc-siRNA binds to the ASGPR expressed by liver hepatocytes and is taken up in endosomes, where the DTM-GalNAc-siRNAs dissociate from the receptors. Then, the DTM-GalNAc sugars and branches are very quickly lysed from the siRNAs before the siRNAs escape to the cytoplasm.

## 3. Materials and Methods

### 3.1. General Information

Unless stated otherwise, commercially available reagents were purchased from Sigma-Aldrich, Acros Organic, Alfa Aesar, TCI, Energy chemical, Adams and Sinopharm Chem. Dry tetrahydrofuran (THF), dichloromethane (DCM), toluene and *N*, *N*-dimethylformamide (DMF) were collected fresh from an Innovative Technology PS-MD-5 solvent purification system. All other dry solvents used were dried over 4 Å molecular sieves and stored under argon. Primary mouse liver cells were purchased from Hunan Fenghui Biotechnology Co., Ltd. (Changsha, China). RNAiMAX was purchased from Invitrogen. Opti-MEM and Paraformaldehyde was purchased from Sigma-Aldrich (Shanghai, China). PBS was purchased from Shanghai Life-iLab Co., Ltd. (Shanghai, China).

All ^1^H NMR, ^13^C NMR spectra were collected using a Bruker nuclear magnetic resonance instrument (300 MHz) using tetramethylsilane (TMS) as the internal standard at room temperature. The ^1^H NMR spectra were referenced to 7.26 ppm in CDCl_3_. Electrospray Ionization Mass spectrometry (ESI-MS) of chemical was performed by electrospray ionization on a 70-VSE. Ultraviolet-Visible Absorption (UV-Vis) of the samples were measured using a Thermo Electron-EV300 UV-Vis spectrophotometer at room temperature. The slit-width was set at 1 nm with a scan speed of 480 nm/min.

### 3.2. Synthesis of DTS Containing GalNAc Conjugate

#### 3.2.1. Synthesis of the DTS Containing GalNAc Scaffold

*Synthesis of compound **2***: In an oven dried round-bottom flask, compound 1 (197 mg, 0.51 mmol), 2-bromoethanol (0.11 mL, 1.54 mmol) and Sc(OTf)_3_ (37 mg, 0.075 mmol as catalyst) were disolved in dry DCM (3 mL). After 27 h, TLC indicated the reaction was complete. The reaction mixture concentrated and directly purified by column chromatography (1:2 PE/EtOAc) to give compound 2 (153 mg, 67 %) as white crystals [38]. ^1^H NMR (300 MHz, CDCl_3_) δ 5.95 (d, *J* = 8.6 Hz, 1H), 5.52–5.12 (m, 2H), 4.77 (d, *J* = 8.4 Hz, 1H), 4.21–4.06 (m, 3H), 4.00–3.89 (m, 2H), 3.86–3.75 (m, 1H), 3.46 (dd, *J* = 9.1, 3.7 Hz, 2H), 2.12 (s, 3H), 2.02 (s, 3H), 1.97 (s, 3H), 1.96 (s, 3H).
*Synthesis of compound **3***: In a 10 mL round-bottom flask with a condenser, compound 2 (454.3 mg, 1.0 mmol) was dissolved in 5.0 mL dry acetone. Then, K_2_CO_3_ (196.5 mg 1.72 mmol) was added. The mixture was heated to 45 °C for 24 h. After cooling to 25 °C, the mixture was filtered. The filter cake was washed with 3 × 10 mL acetone and the combined filtrate was concentrated. The residue was re-dissolved in 15 mL CHCl_3,_ and this solution was washed with 3 × 10 mL water. The organic phase was dried with anhydrous Na_2_SO_4_, and concentrated under a vacuum to afford the crude product as a deep yellow oil, which was purified by flash column on neutral alumina. Eluting with a mixed solvent of PE/EA (*v*/*v* = 4/1 to 1/1) to afford compound 3 (359.6 mg, yield 84%) as a colorless solid [39]. ^1^H NMR (300 MHz, CDCl_3_) δ 5.71 (d, *J* = 8.6 Hz, 1H), 5.47–5.18 (m, 2H), 4.73 (d, *J* = 8.4 Hz, 1H), 4.22–4.08 (m, 2H), 3.97 (dq, *J* = 9.2, 6.4 Hz, 3H), 3.65 (dt, *J* = 10.5, 6.7 Hz, 1H), 3.10 (ddd, *J* = 20.7, 13.8, 7.4 Hz, 2H), 2.35 (s, 3H), 2.15 (s, 3H), 2.05 (s, 3H), 2.01 (s, 3H), 1.99 (s, 3H).
*Synthesis of compound **4***: NaOMe (324.1 mg, 6.0 mmol) was added to a solution of compound **3** (449.5 mg, 1.0 mmol) in CH_3_OH (10 mL) and stirred at RT for 3 h under a nitrogen atmosphere. The mixture was then neutralized using Dowex resin and filtered through a pad of Celite. The collected filtrate was concentrated under reduced pressure and compound 4 (230.7 mg, yield 82 %) was obtained as a foaming oil, which was used without further purification and stored under −20 °C in a nitrogen atmosphere.
*Synthesis of compound **6***: 2,3-dibromomaleimide (5) (254.9 mg, 1.0 mmol) and propargyl bromide (297.4 mg, 2.5 mmol,) were dissolved in acetone (15 mL), followed by the addition of potassium carbonate (552.8 mg, 4.0 mmol). The mixture was stirred at 25 °C for 48 h. The precipitate was filtrated off and the filtrate was concentrated to afford the crude product, which was purified by the silica gel column chromatography using hexane/ethyl acetate (1/1, *v*/*v*) as the eluent to obtain the desire product (158.2 mg, yield 54.0%) [34]. ^1^H NMR (300 MHz, CDCl_3_) δ 4.38 (d, *J* = 2.5 Hz, 2H), 2.27 (s, 1H).
*Synthesis of compound **7***: In a 25 mL round-bottom flask under argon, compound **6** (290.9 mg, 1.0 mmol) was dissolved in MeOH (4.0 mL). Then, NaOAc (172.3 mg, 2.1 mmol) was added to the mixture. Then, a solution of compound **4** (618.9 mg, 2.2 mmol) in MeOH (2 mL) under argon was added dropwise over 5 min, to give an orange solution. After that, the mixture was stirred at 20 °C for 3 h. Then, quenched with 10 mL H_2_O and extracted with EtOAc (2 × 20 mL). The combined organic layer was dried (MgSO_4_), filtered and concentrated under a vacuum to yield a yellow solid which was used without any purification [40].

#### 3.2.2. Synthesis of DTS Containing GalNAc Conjugated and Unconjugated siRNAs

Sense and antisense strands (Table 1) were synthesized on an ABI394 Synthesizer (Applied Biosystems, Foster City, CA, USA) using commercially available 5′-*O*-(4,4′-dimethoxytrityl)-2′-deoxy-2′-fluoro- and 5′-*O*-(4,4′-dimethoxytrityl)-2′-*O*-methyl- 3′-*O*-(2-cyanoethyl-*N*,*N*-diisopropyl) phosphoramidite monomers of uridine, 4-*N*-acetylcytidine, 6-*N*-benzoyladenosine, and 2-*N*-isobutyrylguanosine (Hongene Biotech Corporation, Shanghai, China) using standard solid-phase oligonucleotide synthesis and deprotection protocols. After synthesis, the support was treated with 25–28% aqueous ammonia at 55 °C for 16 h. The suspension was filtered through a 0.22 μm filter to remove solid residues. The oligonucleotides were purified by an anion-exchange high-performance liquid chromatography (IEX-HPLC) with Source 15Q (Cytiva, Shrewsbury, MA, USA), using a linear gradient of 15–65% buffer B over 50 min with a 15 mL/min flow rate. Buffer A was 0.02 M Na_2_HPO_4_ in 10% CH_3_CN (pH 8.5) and buffer B was buffer A plus 1 M NaCl. The pure fractions were combined, concentrated and desalted on a Sartorius ultrafiltration station. The integrities of the purified oligonucleotides were confirmed by LC-MS (Table S1), and by analytical IEX HPLC. Equimolar amounts of complementary sense and antisense strands were mixed and annealed by heating to 90 °C and slowly cooling to obtain the desired siRNAs (Table 1).

According to Figure 1, Oligo-2NH_2_ was synthesized by oligo-NH_2_ reacting with 2NH_2_-NHS Ester (10-fold excess over oligo-NH_2_) in the mixture of DMSO and phosphate-buffered saline (PBS) (3:2, *v*/*v*) at 25 °C overnight. The mixture was filtered through a 0.22 μm filter and purified by RP-HPLC with Waters X-Bridge C18 column (5 μm, 10 mm × 250 mm) using a linear gradient of 5–45% buffer B over 36 min with a 5 mL/min flow rate (Buffer A: 0.1 M TEAA (pH 7.0), Buffer B: ACN). The Oligo-2NH_2_ was characterized by RP-HPLC and electrospray ionization mass spectrometry (ESI-MS), (Figure 2).

Oligo-2N_3_ was synthesized by oligo-2NH_2_ reacting with the N_3_-NHS Ester (50-fold excess over oligo-NH_2_) in the mixture of DMSO and phosphate-buffered saline (PBS) (1:3, *v*/*v*) at 4 °C overnight. The mixture was filtered through a 0.22 μm filter and purified by RP-HPLC with Waters X-Bridge C18 column (5 μm, 10 mm × 250 mm) using a linear gradient of 5–60% buffer B over 40 min with 5 mL/min flow rate (Buffer A: 0.1 M TEAA (pH 7.0), Buffer B: ACN). Oligo-2N_3_ was characterized by RP-HPLC and electrospray ionization mass spectrometry (ESI-MS) (Figure 2).

DTM-GalNAc-siRNA was synthesized by Oligo-2N_3_ reacting with 2Gal-DTM-alkyne (12-fold excess over oligo-2N_3_) in the presence of an excess of CuSO_4_·5H_2_O (12-fold excess over oligo-2N_3_), THPTA (30-fold excess over oligo-2N_3_) and sodium ascorbate (50-fold excess over oligo-2N_3_) in a PBS/DMF (1:1, *v*/*v*) solvent mixture at room temperature for 1 h. The mixture was filtered through a 0.22 μm filter and purified by RP-HPLC with Waters X-Bridge C18 column (5 μm, 10 mm × 250 mm) using a linear gradient of 5–60% buffer B over 55 min with 5 mL/min flow rate (Buffer A: 0.1 M TEAA (pH 7.0), Buffer B: ACN). DTM-GalNAc-siRNA was characterized by RP-HPLC and electrospray ionization mass spectrometry (ESI-MS) (Figure 2). Equimolar amounts of complementary sense and antisense strands were mixed and annealed by heating it to 90 °C and slowly cooling it to obtain the desired siRNAs (Table 1).

## 4. Conclusions

In summary, we have developed a novel DTM-based tetra-antennary GalNAc conjugate, which could be readily fabricated by three efficient click-type reactions, that is, amidations, thiol-dibromomaleimide addition and CuAAC. With this DTM GalNAc scaffold, a siRNA delivery efficiency comparable with the GalNAc L96 standard to mTTR target was achieved. Additionally, due to the internal DTMs, a highly fluorescent emission was observed, which would benefit the delivery tracking and avoid the incorporation of extra hydrophobic fluorophores. The insertion of DTMs into GalNAc conjugate might seem trivial, but this two-birds-with-one-stone strategy has potential applications in gene and drug delivery in medical therapy.

## Figures and Tables

**Table 1:** siRNA used in this study.

siRNA	S/AS ^a^ (Sequence 5′-3′) ^b^	Target ^c^
1	CmsAmsGmUmGfUmUfCfUfUmGmCmUmCmUmAmUmAmAm/ UmsUfsAmUmAmGfAmGmCmAmmGmAmAfCmAfmUmGmsUmsUm	*mTTR*
2	CmsAmsGmUmGfUmUfCfUfUmGmCmUmCmUmAmUmAmAm/ UmsUfsAmUmAmGfAmGmCmAmmGmAmAfCmAfmUmGmsUmsUm (FAM)	

^a^ S and AS represent sense and antisense strands, respectively. ^b^ Upper-case, italicized upper-case, and lower-case letters indicate 2′-OH, 2′-deoxy-2′-fluoro, and 2′-*O*-methyl sugar modifications, respectively, toadenosine (A), cytidine (C), guanosine (G), and uridine (U). ^c^ siRNAs target mRNAs encoding mouse transthyretin (mTTR).

## Data Availability

Raw data are available from the authors upon request.

## Supplementary Materials

The following supporting information can be downloaded at: https://www.mdpi.com/article/10.3390/molecules28207184/s1. Scheme S1: Synthetic route of compound **7** (2Gal-DTM-alkyne); Figure S1: ^1^H NMR spectrum of compound **2**; Figure S2: ^1^H NMR spectrum of compound **3**; Figure S3: ^1^H NMR spectrum of compound **4**; Figure S4: ^1^H NMR spectrum of compound **6**; Figure S5: ^1^H NMR spectrum of compound **7**.

## Sample Availability

Not available.
