# Supplementary material for: Two Birds with One Stone: A Novel Dithiomaleimide-Based GalNAc-siRNA Conjugate Enabling Good siRNA Delivery and Traceability
Source: Molecules. 2023 Oct 19;28(20):7184. doi: 10.3390/molecules28207184 (PMC10609014; doi:10.3390/molecules28207184)
Supplement: Supplementary file 1 [file molecules-28-07184-s001.zip › molecules-2625639-supplementary.pdf]

Supplementary Information

Table of Contents.. **Error! Bookmark not defined.**

Experimental Procedures 2

1. Synthesis of the DTS containing GalNAc scaffold 2

2. NMR spectra of the intermeidates 3

3. In vitro free-uptake of siRNA-GalNAc conjugate compared with L96 5

Experimental Procedures

1. Synthesis of the DTS containing GalNAc scaffold

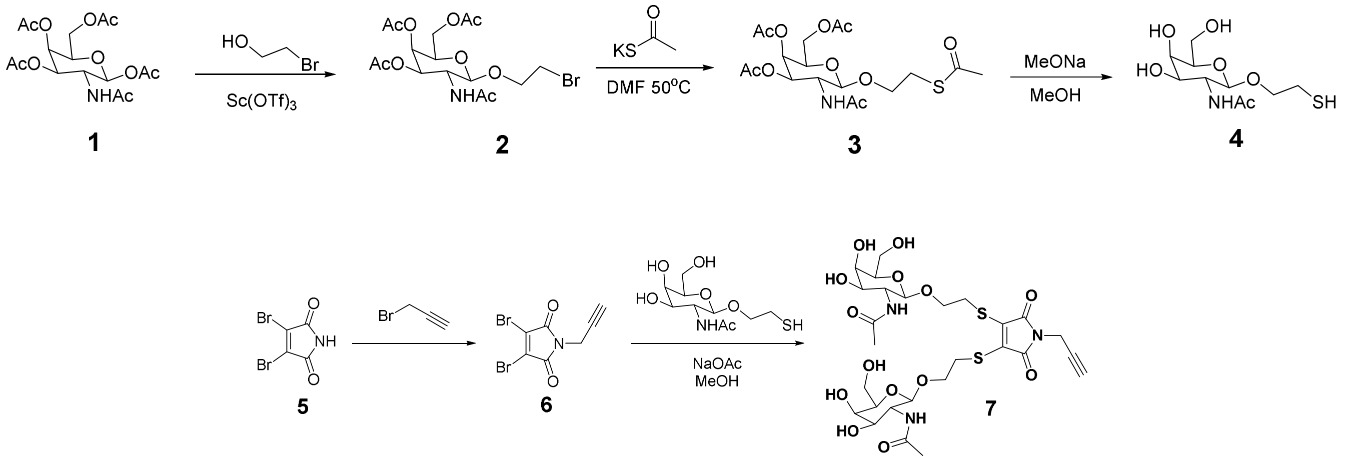

Scheme S1. Synthetic route of compound **7** (2Gal-DTM-alkyne).

2. NMR spectra of the intermeidates

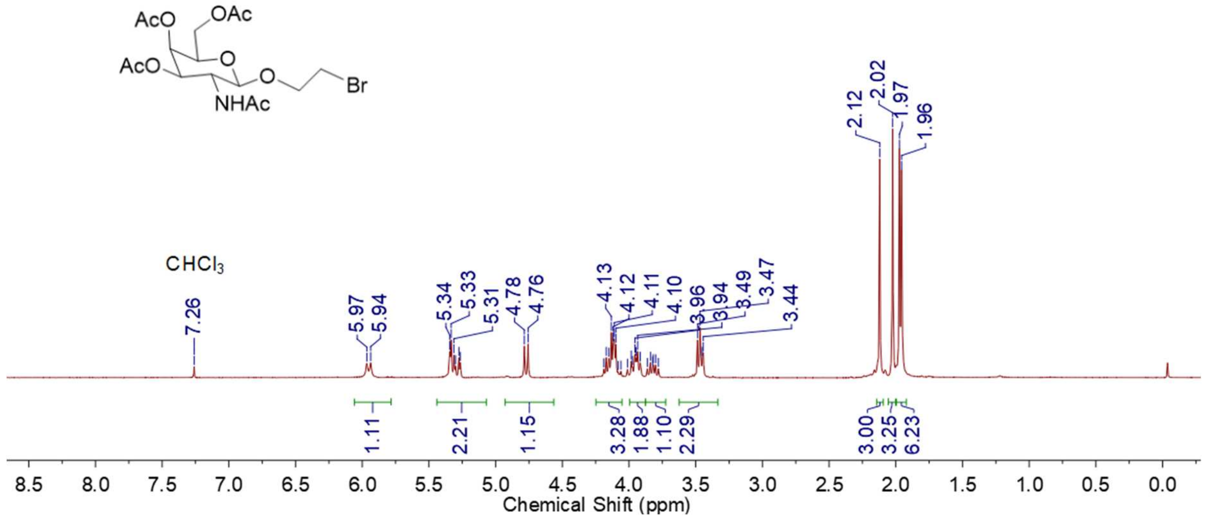

Figure S1. ^{1}H NMR spectrum of compound **2** in $CDCl_3$ (Bruker, 300 MHz, TMS)

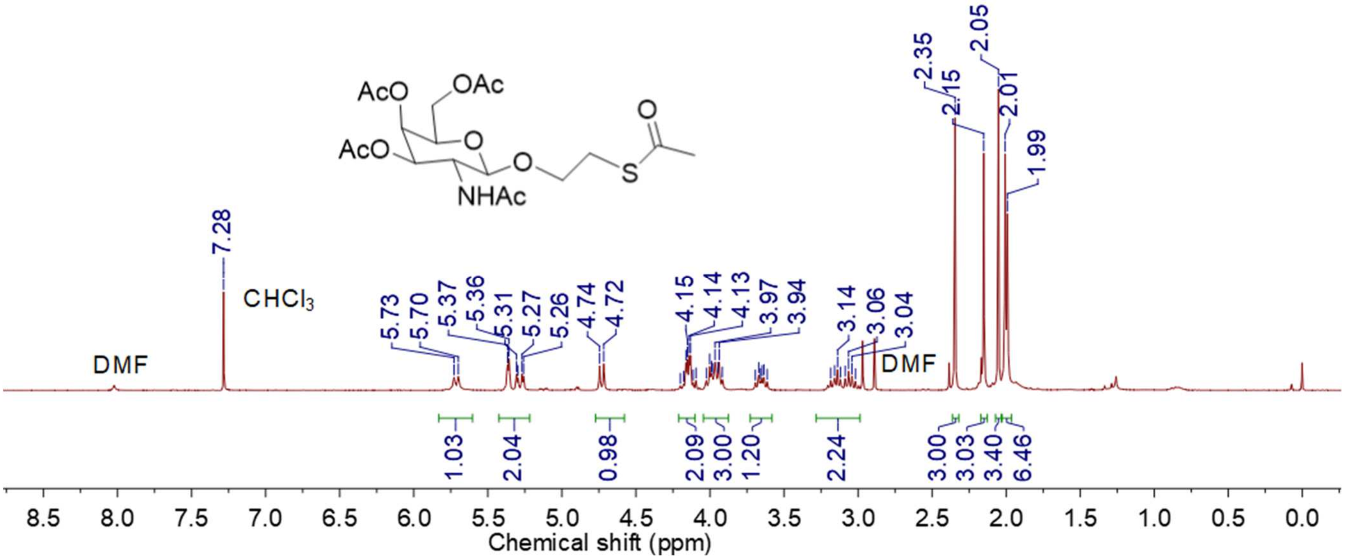

Figure S2. ^{1}H NMR spectrum of compound **3** in $CDCl_3$ (Bruker, 300 MHz, TMS)

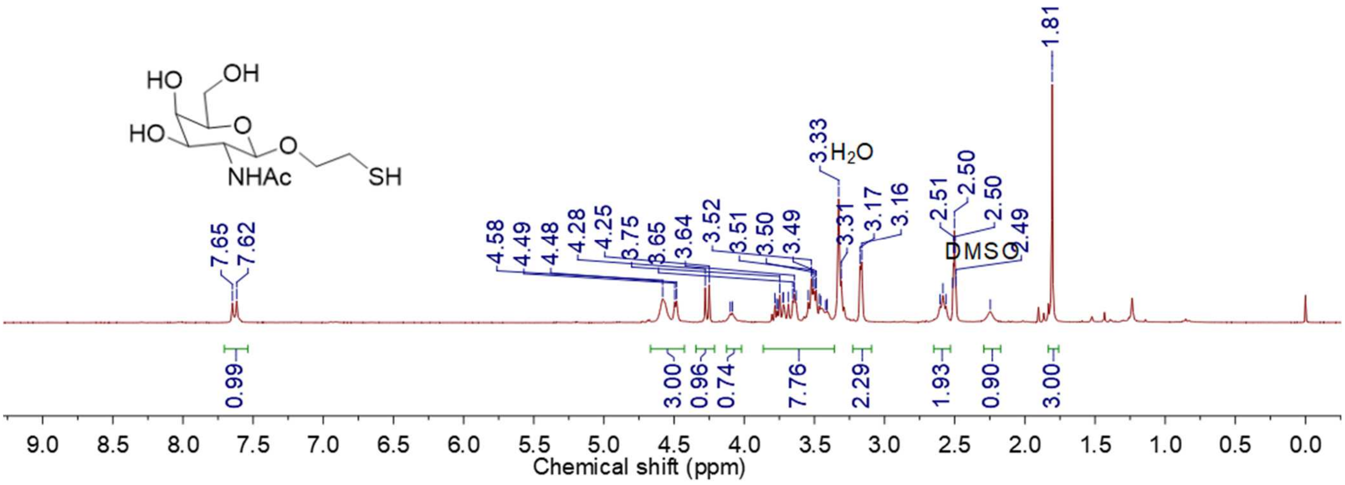

Figure S3. ^{1}H NMR spectrum of compound **4** in DMSO-d_6 (Bruker, 300 MHz, TMS)

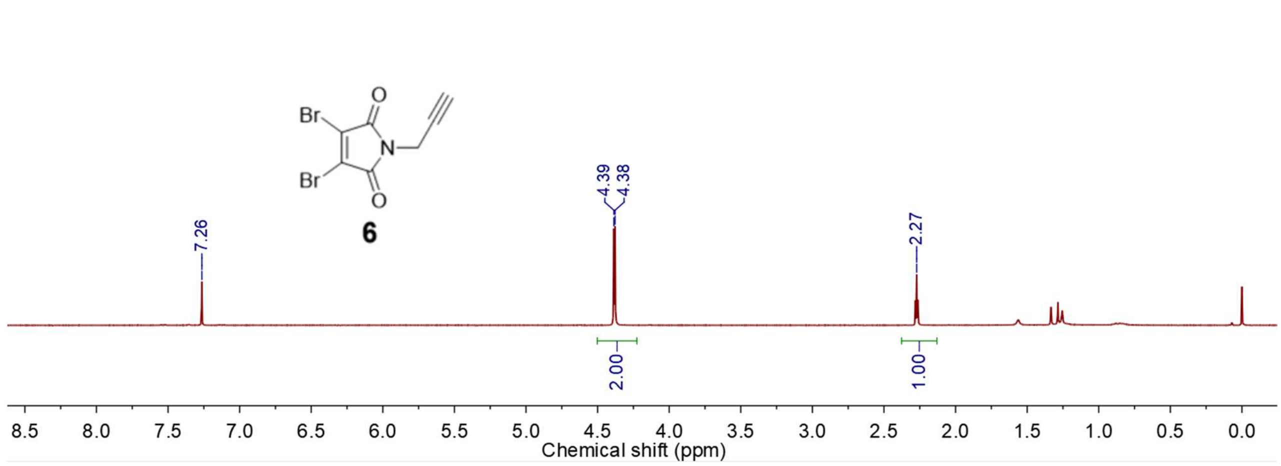

Figure S4. ^{1}H NMR spectrum of compound **6** in $CDCl_3$ (Bruker, 300 MHz, TMS)

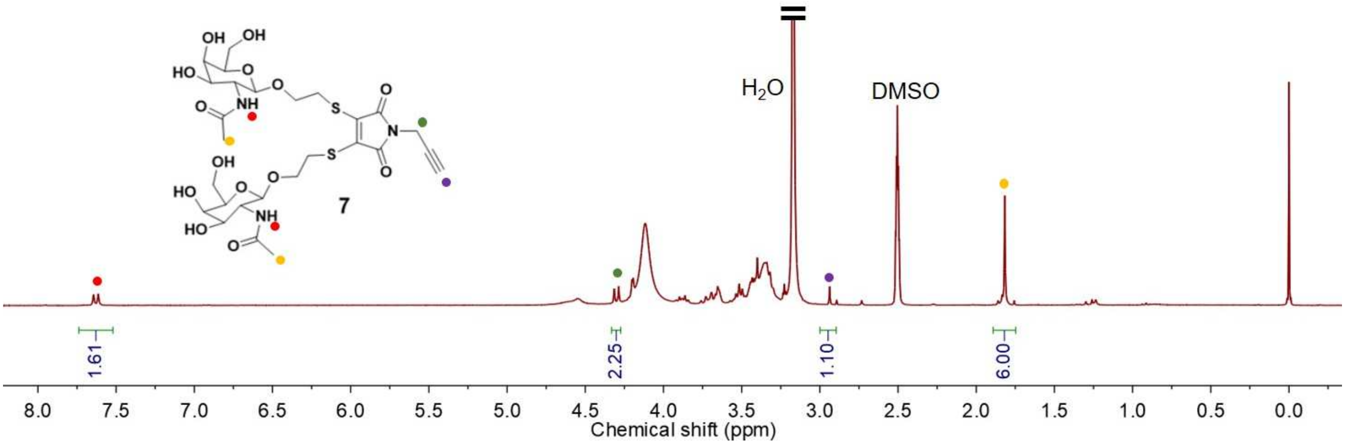

Figure S5. ^{1}H NMR spectrum of compound compound **7** in DMSO-d_6 (Bruker, 300 MHz, TMS)

3. In vitro free-uptake of siRNA-GalNAc conjugate compared with L96

Primary mouse liver cells were isolated and transfected in 96-well plates with 8000 cells per well. Afterwards, transfection samples were divided into two groups: Free-uptake group and RNAiMAX group. In the free-uptake group, a maximum concentration of 10nM was selected for 4-fold dilution with a total of 8 concentrations, while in the transfection group, a maximum concentration of 0.1nM was selected for 10-fold dilution with a total of 3 concentrations for quality control. After transfection, they were cultured in cell culture box. After 12 h the data is exported after the reading of each hole and the residual suppression efficiency of each sample is calculated. Plot was summary using GraphPad Prism 7 software. For CLSM studies, Primary mouse liver cells were seeded into 24-well culture plates at a density of 5.0×10^4 cells per well and cultured overnight. Then the medium was replaced with medium containing DTM-GalNAc and GalNAc-L96 (FAM) in Opti-MEM (Sigma-Aldrich) for 2 h, respectively. Afterwards，the medium was removed and the cells were washed by cold PBS buffer before fixed with 4% paraformaldehyde. Subsequently, cell nuclei were stained with PI. All slices were observed with a laser scanning confocal microscope (Leica TCS SP8).
